# Notch Activation Differentially Regulates Renal Progenitors Proliferation and Differentiation Toward the Podocyte Lineage in Glomerular Disorders

**DOI:** 10.1002/stem.492

**Published:** 2010-08-02

**Authors:** Laura Lasagni, Lara Ballerini, Maria Lucia Angelotti, Eliana Parente, Costanza Sagrinati, Benedetta Mazzinghi, Anna Peired, Elisa Ronconi, Francesca Becherucci, Daniele Bani, Mauro Gacci, Marco Carini, Elena Lazzeri, Paola Romagnani

**Affiliations:** a Excellence Centre for Research, Transfer and High Education for the Development of DE NOVO Therapies (DENOTHE), University of FlorenceFlorence, Italy; b Department of Anatomy, Histology and Forensic Medicine, University of FlorenceFlorence, Italy; c Department of Medical and Surgical Critical Care, University of FlorenceFlorence, Italy; d Pediatric Nephrology Unit, Meyer University HospitalFlorence, Italy

**Keywords:** Renal stem cells, Renal progenitors, Glomerulosclerosis, Kidney, Glomerulonephritis

## Abstract

Glomerular diseases account for 90% of end-stage kidney disease. Podocyte loss is a common determining factor for the progression toward glomerulosclerosis. Mature podocytes cannot proliferate, but recent evidence suggests that they can be replaced by renal progenitors localized within the Bowman's capsule. Here, we demonstrate that Notch activation in human renal progenitors stimulates entry into the S-phase of the cell cycle and cell division, whereas its downregulation is required for differentiation toward the podocyte lineage. Indeed, a persistent activation of the Notch pathway induced podocytes to cross the G_2_/M checkpoint, resulting in cytoskeleton disruption and death by mitotic catastrophe. Notch expression was virtually absent in the glomeruli of healthy adult kidneys, while a strong upregulation was observed in renal progenitors and podocytes in patients affected by glomerular disorders. Accordingly, inhibition of the Notch pathway in mouse models of focal segmental glomerulosclerosis ameliorated proteinuria and reduced podocyte loss during the initial phases of glomerular injury, while inducing reduction of progenitor proliferation during the regenerative phases of glomerular injury with worsening of proteinuria and glomerulosclerosis. Taken altogether, these results suggest that the severity of glomerular disorders depends on the Notch-regulated balance between podocyte death and regeneration provided by renal progenitors. Stem Cells 2010; 28:1674–1685.

## INTRODUCTION

Glomerular diseases account for 90% of end-stage kidney disease [1]. Depletion of podocytes, which are highly specialized cells that are critical constituents of the glomerular filtration barrier, is key for progression of glomerular disorders toward glomerulosclerosis [2–5]. However, converging evidence demonstrated that in adult kidneys podocytes can get replaced from a resident population of progenitors localized along the inner surface of the Bowman's capsule [6–11]. These renal progenitors can generate podocytes via their division and migration along the Bowman's capsule toward the glomerular tuft, where complete differentiation into podocytes occurs [9–12]. In humans, renal progenitors are characterized by the presence of surface markers, CD133 and CD24 [6–10,12]. Understanding of how self-renewal and fate decision of renal progenitors may be perturbed in pathological conditions is of crucial importance. Indeed, recent results demonstrated that abnormal proliferation of renal progenitors can generate glomerular lesions in focal segmental glomerulosclerosis (FSGS) and crescentic glomerulonephritis [13,14], suggesting that the response of renal progenitors to podocyte injury is critical to achieve remission or progression of glomerular disorders [15,16]. However, the mechanisms that regulate the growth and differentiation of renal progenitors are still unknown.

The pathways involved in stem/progenitor cell function are mostly conserved in adult organs [17]. In particular, the Notch pathway affects cell fate choices of stem/progenitor cells, including the decisions to self-renew or differentiate [17–21]. In mammals, there are four single-pass transmembrane Notch receptors (Notch1/2/3/4) and five transmembrane ligands, Delta-like (Dll)-1/3/4, Jagged (Jag)-1/2. On activation, the Notch intracellular domain (NICD) is cleaved by γ-secretases and translocates into the nucleus to interact with the transcriptional regulator recombination signal-binding protein-J (RBP-J) [17–21], then activates the downstream transcriptional targets such as the Hairy enhancer of split (Hes) factors and Hes-related repressor proteins (Hey) [17–22].

In this study, the role of Notch signaling in the growth/differentiation of renal progenitors toward the podocyte lineage was investigated. We found that activation of the Notch pathway in human renal progenitors stimulated their entry into the S-phase of the cell cycle and cell division, while a downregulation of the Notch pathway was required for their differentiation toward the podocyte lineage. Indeed, persistent activation of the Notch pathway induced podocytes to cross the cell cycle G_2_/M checkpoint, resulting in cytoskeleton disruption and death by mitotic catastrophe. Notch activation was virtually undetectable in glomeruli of healthy adult kidneys, but it was observed in renal progenitors and podocytes of patients affected by glomerular disorders. Accordingly, inhibition of the Notch pathway in mice models of FSGS ameliorated proteinuria and reduced podocyte death during the initial phases of glomerular injury, whereas it induced worsening of proteinuria and reduction of renal progenitor proliferation during the regenerative phases of chronic glomerular injury. Taken altogether, these results suggest that a regulated activation of the Notch pathway in renal progenitors during their phases of differentiation toward the podocyte lineage is critical to balance injury and regeneration in glomerular disorders.

## MATERIALS AND METHODS

### Patients

We studied kidney biopsy specimens of patients with systemic lupus erythematosus (LES) nephritis (*n* = 15) and FSGS (*n* = 10). Normal renal tissues were obtained from patients (*n* = 8) nephrectomized because of renal cell carcinoma, in agreement with the Ethical Committee on human experimentation of the Azienda Ospedaliero-Universitaria Careggi, Florence.

### Human Renal Progenitor Cultures and In Vitro Differentiation

Human renal progenitors were obtained and cultured as previously described [6–10]. For podocyte differentiation, cells were treated for 2 days with vitamin D_3_, retinoic acid and dexamethasone-supplemented DMEM/F12 (VRADD) medium composed of Dulbecco(s modified Eagle medium (DMEM)-F12 (Sigma, ST. Louis, MO, USA, http://www.sigmaaldrich.com) supplemented with 10% fetal bovine serum (FBS) (Hyclone, from Euroclone, Milan, Italy; http://www.euroclonegroup.it), 100 nM vitamin D3, 100 μM *all-trans* retinoic acid and 0.1 μM Dexamethasone (all from Sigma).

### Cell Proliferation Assay

^3^[H]-thymidine incorporation was assessed as described [23]. The γ-secretase inhibitor IX DAPT (N-[N-(3,5-Difluorophenacetyl)-L-alanyl]-S-phenylglycine t-butyl ester) was obtained from Merck, KGaA, Darmstadt, Germany (http://www.merck.com).

### Human Renal Progenitor Infection

N1ICD (5275–7332 bp), N2ICD (5094–7413 bp), or N3ICD (4954–6996 bp) were polymerase chain reaction (PCR)-amplified from renal progenitor cells cDNA and cloned into the bicistronic pLVX-IRES-sGreen1 lentiviral vector (Clontech, Mountain View, CA, USA, http://www.clontech.com) leading to the gene-of-interest and the ZsGreen1 fluorescent protein to be simultaneously coexpressed from a single mRNA transcript. Lentiviral particles were produced by cotransfection of the lentiviral and the three third-generation packaging vectors into Lenti-X 293T cells (Clontech). Titers were determined by infection of 293T cells and ranged from 0.2 × 10^6^ to 2 × 10^6^ infectious particles per milliliter.

Human renal progenitors were infected with virus-containing supernatant with a multiplicity of infection (MOI) of 10 as well as fresh endothelial cell growth medium-microvascular (EGM-MV) media, in the presence of 8 μg/ml of polybrene (Sigma). Level of infection was assessed by measuring percentages of ZsGreen1+ cells by flow cytometry. Percentages of infected cells ranged from 40% to 90%.

### RBP-J Reporter System

pGreenFire1 (pGF1)-Notch reporter plasmid, which expresses firefly luciferase reporters under the control of RBP-J and the control empty vector, were obtained from System Biosciences (SBI, Mountain View, CA, USA, http://www.systembio.com). Lentiviral particles were produced as described earlier and human renal progenitors were infected with a MOI of 30.

### Cell Cycle Analysis and Propidium Iodide/Annexin V Staining

Cell cycle analysis was performed as previously described and analyzed with the Modfit LT 3.0 software (Verity Software House Inc., Topsham, ME, USA, http://www.vsh.com) [23]. Apoptosis and/or necrosis were evaluated using propidium iodide (PI) and the Annexin V kit (BD Biosciences, San Diego, CA, USA, http://www.bdbiosciences.com) and the FACSDiva software.

### Immunofluorescence and Confocal Microscopy

Confocal microscopy was performed on 5 μm sections of renal frozen tissues or on cells by using a LSM510 META laser confocal microscope (Carl Zeiss, Jena, Germany, http://www.zeiss.com) as described previously [6–8]. The following antibodies were used: anti-Notch1 pAb Val1744 (recognizing NICD), anti-Notch3 pAb (recognizing NICD), anti-Dll4 pAb (all from Abcam, Cambridge, U.K., http://www.abcam.com); Notch2 pAb (recognizing NICD, Rockland, Gilbertsville, PA, USA, http://www.rockland-inc.com); anti-Notch1 pAb C-20 (recognizing NICD), anti-Jag1 pAb, anti-Jag2 pAb, anti-nephrin pAb, anti-CD24 mAb (clone SN3), anti-WT1 mAb (clone F-6; all from Santa Cruz Biotechnologies, Santa Cruz, CA, USA, http://www.scbt.com); anti-Hes1 pAb (Chemicon, Temecula, CA, USA, http://www.chemicon.com); anti-Dll1 mAb (clone 251123, R&D Systems, Minneapolis, MN, USA, http://www.rndsystems.com), anti-H3-Ser10 mAb (anti-phospho histone H3, Abcam); anti-Claudin-1 mAb (clone 2H10D10, Invitrogen, Carlsbad, CA, USA, http://www.invitrogen.com); anti-H3-Ser10 pAb (Cell Signaling, Danvers, MA, USA, http://www.cellsignal.com), anti-α-tubulin mAb (clone B512, Sigma); anti-podocalyxin (PDX) mAb (clone 222328, R&D Systems); anti-cytokeratin mAb (CK, clone C-2562, Sigma). Staining with Alexa Fluor546 phalloidin (Molecular Probes, Invitrogen) was performed following manufacturer's instructions. Double immunolabeling was performed as described and Alexa-Fluor secondary antibodies were obtained from Molecular Probes.

### Analysis of Renal Morphology and Quantification of Nephrin Positive Cells and Mitotic Cells

Five micrometer-thick kidney sections of mice treated with vehicle and DAPT, killed at days 7 and 21, were analyzed. For quantitation of podocytes or mitotic podocytes, the number of nephrin positive cells or of H3-Ser10/Nephrin double positive cells was evaluated in 15 glomeruli of at least four sections for each mouse by two independent observers. For quantitation of mitotic renal progenitors, the number of H3-Ser10/Claudin-1 double positive cells was evaluated by two independent observers in at least six sections for each mouse.

### Transmission Electron Microscopy

Kidney tissue samples of about 2 mm^3^ obtained from four DAPT-treated mice and four oil-treated mice were fixed in glutaraldehyde-osmium tetroxide, embedded in epoxy resin and routinely processed for transmission electron microscopical observation. Ultrathin sections were cut using a LKB-Nova ultramicrotome (LKB, Bromma, Sweden, http://www.lkb.com), counterstained with uranyl acetate and alkaline bismuth subnitrate, and examined under a JEM 1010 transmission electron microscope (Jeol, Tokyo, Japan, http://www.jeol.com) at 80 kV.

### Real-Time Quantitative Reverse Transcription PCR

Taq-Man reverse transcription (RT)-PCR was performed as previously described [24]. Notch1, Notch2, Notch3, Notch4, Dll1, Dll3, Dll4, Jag1, Jag2, Hes1, Hes2, Hes3, Hes4, Hes5, Hes6, Hes7, Hey1, Hey2, HeyL, nephrin, p21, p27, and Aurora kinase B quantification was performed using Assay on Demand kits (Applied Biosystems, Warrington, U.K., http://www.appliedbiosystems.com).

### Severe Combined Immunodeficient (SCID) Mouse Model of Adriamycin Nephropathy

Animal experiments were performed in accordance with institutional, regional, and state guidelines and in adherence to the National Institutes of Health Guide for the Care and Use of Laboratory Animals. Adriamycin nephropathy was induced in female SCID mice (Harlan, Udine, Italy, http://www.harlan.com) at the age of 6 weeks by a single i.v. injection of adriamycin (6 mg/kg in phosphate buffered saline (PBS), Sigma) on day 0 in the tail vein (*n* = 12) in a total of three independent experiments. As controls, another group of mice (*n* = 12) received PBS. Proteinuria was evaluated on day 0 and again on days 7, 14, 21, 28, 35, and 42 in adriamycin or PBS-treated SCID mice. In each of the same experiments, 28 additional mice treated as described earlier were killed at day 0 (*n* = 4), 7 (*n* = 4), 14 (*n* = 4), 21 (*n* = 4), 28 (*n* = 4), 35 (*n* = 4), and 42 (*n* = 4) for analysis of renal morphology and Notch immunofluorescence.

Additional groups of mice received oral gavage of DAPT (5 mg/kg in corn oil, Calbiochem, Merck) or of vehicle (corn oil) once a day from 2 days before the day of adriamycin injection to the end of the experiment, as follows: group 1, vehicle (*n* = 15); group 2, DAPT (*n* = 15) in a total of three independent experiments. In each of the same experiments, 20 additional mice were killed at day 7 (*n* = 5), 14 (*n* = 5), 21 (*n* = 5), and 28 (*n* = 5) after adriamycin injection for analysis of renal morphology. Urinary albumin and creatinine in 24 hours urine were determined with Albuwell M kit (Exocell, Philadelphia, PA, USA, http://www.exocell.com) and Creatinine Assay kit (Cayman Chemical, Ann Arbor, MI, USA, http://www.caymanchem.com).

### Statistical Analysis

The results were expressed as mean ± SEM.Comparison between groups was performed by the Mann-Whitney test, the Wilcoxon test, or through the analysis of variance for multiple comparisons (ANOVA; ANOVA for repeated measures). *p* < .05 was considered to be statistically significant.

## RESULTS

### Notch Activation Induces Proliferation of Renal Progenitors

**Figure 1:**
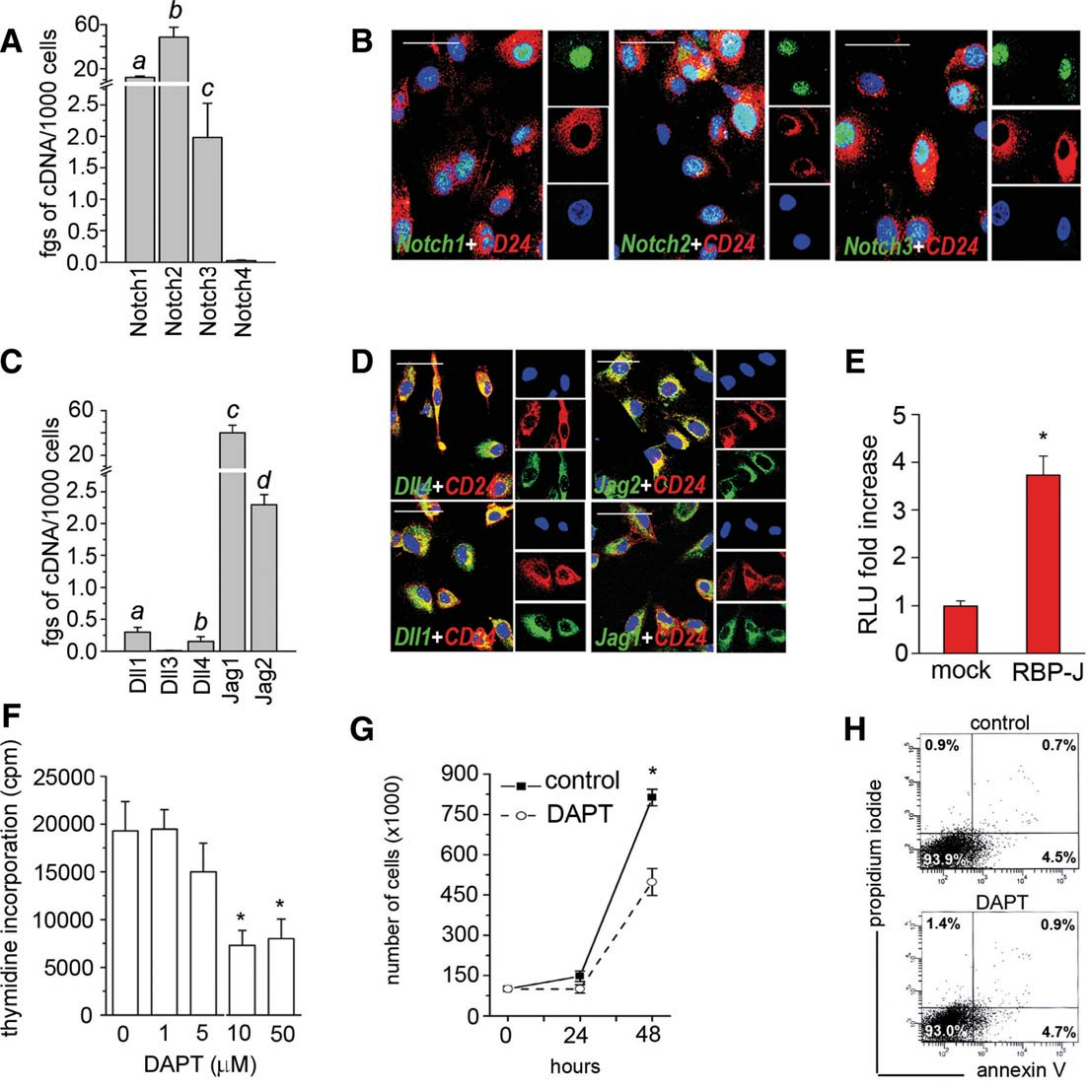
Expression and functional properties of Notch receptors and ligands on human renal progenitors. (**A**): mRNA levels for Notch receptors as assessed by real-time quantitative reverse transcription polymerase chain reaction (RT-PCR) in cultures of renal progenitor cells. Results are expressed as mean ± SEM of triplicate assessments in primary cultures from five different donors. *b* versus *a*, *c* and *a* versus c: *p* < .05. (**B**): Double label immunofluorescence for Notch1, Notch2, or Notch3 (green) and CD24 (red) in renal progenitors, as assessed by confocal microscopy. Topro-3 (blue) counterstains nuclei. One representative of seven independent experiments is shown. Scale bar = 50 μm. (**C**): mRNA levels for Notch ligands as assessed by real-time quantitative RT-PCR in cultures of renal progenitors. Results are expressed as mean ± SEM of triplicate assessments in primary cultures from five different donors. *c* versus *a*, *b*, *d* and *d* versus *a*, *b*: *p* < .05. (**D**): Double label immunofluorescence for Dll1, Dll4, Jag1, or Jag2 (green) and CD24 (red) in renal progenitors, as assessed by confocal microscopy. Topro-3 (blue) counterstains nuclei. One representative of seven independent experiments is shown. Scale bar = 50 μm. (**E**): Activation of the Notch pathway in renal progenitors as assessed by infection with a RBP-J-responsive luciferase vector. Results are expressed as mean ± SEM of relative luciferase units in comparison with human renal progenitors infected with the empty vector as obtained in nine independent experiments. (*, *p* < .05). (**F**): Effect of DAPT (0–50 μM) treatment on the proliferative capacity of renal progenitors, as assessed by ^3^[H]-thymidine incorporation. Results represent mean values ± SEM obtained in four separate experiments from five different donors (*, *p* < .05). (**G**): Growth curves showing the effect of DAPT treatment on renal progenitor numbers. Results are expressed as mean ± SEM of cell numbers obtained in four separate experiments from five different donors (*, *p* < .05). (**H**): FACS analysis of renal progenitor cell viability following their treatment with DAPT, as assessed by annexin-V, and propidium iodide staining. One representative of four experiments is shown. Abbreviations: DAPT, N-[N-(3,5-Difluorophenacetyl)-L-alanyl]-S-phenylglycine t-butyl ester; RBP-J, recombination signal-binding protein-J; FACS, fluorescence activated cell sorting, RLU, relative luminescence unit.

First of all, we examined the mRNA and protein expression profiles of Notch ligands and receptors in cultured human renal progenitor cells. Renal progenitors expressed Notch1, Notch2, and Notch3, whereas expression of Notch4 was irrelevant (Fig. 1A, 1B). Notch2 mRNA levels were significantly higher than those of Notch1 and Notch3 (*p* < .05). Among Notch ligands, Dll1, Dll4, Jag1 and Jag2 were all present in human renal progenitors. Jag1 exhibited the highest expression level (*p* < .05), and Jag2 was significantly more expressed than Dll1 and Dll4 (*p* < .05; Fig. 1C, 1D), whereas Dll3 was not detectable (Fig. 1C). Detection of Notch1, Notch2, and Notch3 proteins within nuclei using antibodies against the NICDs suggested that the Notch signaling pathway was active in most renal progenitors. Accordingly, infection with a RBP-J-responsive luciferase vector induced activation of the RBP-J-dependent promoter reporter, further confirming that the Notch pathway was chronically active in human renal progenitors culture (Fig. 1E).

To investigate the role of Notch signaling on renal progenitor proliferation, cells were exposed to different concentrations of the γ-secretase inhibitor DAPT, which inhibits the signaling pathway of all Notch receptor types. Assessment of ^3^H-thymidine incorporation demonstrated that DAPT-treatment induced a dose-dependent growth inhibition (Fig. 1F), which resulted in a reduction of renal progenitor numbers (Fig. 1G). Viability of 10 μM DAPT-treated and DAPT-untreated cells was comparable (94.6% ± 0.7% vs. 93.6% ± 0.9%; *NS*), as evaluated by using a combined fluorescence activated cell sorting (FACS) analysis for PI and annexin V staining (Fig. 1H), thus demonstrating that inhibition of the Notch pathway was not associated with a reduction in renal progenitor viability.

### Notch Downregulation Is Necessary to Achieve Differentiation of Human Renal Progenitors into the Podocyte Lineage

**Figure 2:**
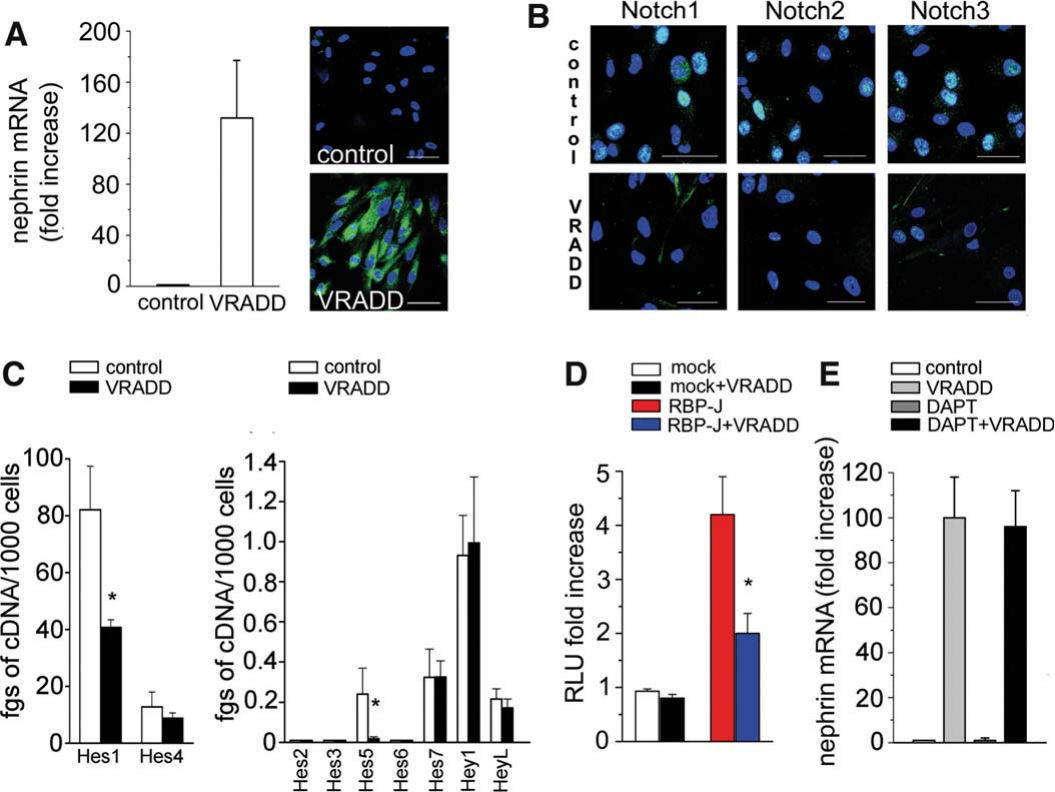
Notch pathway downregulation during human renal progenitors differentiation toward the podocyte lineage. (**A**): Left: Nephrin mRNA levels in renal progenitors before and after culture in VRADD medium, as assessed by real time quantitative reverse transcription polymerase chain reaction (RT-PCR). Results are expressed as mean ± SEM of fold level increase for nephrin mRNA before (control) and after 48 hours of culture in VRADD medium obtained in four separate experiments. Right: Nephrin protein expression (green) in renal progenitors as assessed by confocal microscopy before (top) and after (bottom) 48 hours of culture in VRADD medium. Topro-3 (blue) counterstains nuclei. One representative of four independent experiments is shown. Scale bar = 50 μm. (**B**): Notch1, Notch2, and Notch3 protein expression (green) as assessed by confocal microscopy in control cells (top) and after 48 hours of culture in VRADD medium (bottom). Topro-3 (blue) counterstains nuclei. One representative of eight independent experiments is shown. Scale bar = 50 μm. (**C**): Notch-target genes mRNA levels in renal progenitors before (control) and after 48 hours of culture in VRADD medium, as assessed by real time quantitative RT-PCR. Results are expressed as mean ± SEM obtained in six separate experiments. (*, *p* < .05). (**D**): Downregulation of the Notch pathway activity in renal progenitors following differentiation toward the podocyte lineage, as assessed by infection with a reporter vector for the RBP-J transcriptional response element. Results are expressed as mean ± SEM fold increase of luciferase activity in comparison with renal progenitors infected with the empty expression vector as obtained in eight independent experiments. (*, *p* < .05). (**E**): Effect of DAPT treatment on the increase of mRNA level for nephrin in renal progenitors before (control) and after 48 hours of culture in VRADD medium. Results are expressed as mean ± SEM of fold level increase obtained in four separate experiments. Abbreviations: DAPT, N-[N-(3,5-Difluorophenacetyl)-L-alanyl]-S-phenylglycine t-butyl ester; fgs, femtograms; RBP-J, Recombination signal-binding protein-J; RLU, relative luminescence unit; VRADD, vitamin D_3_, retinoic acid and dexamethasone-supplemented DMEM/F12.

In agreement with previous studies [10], culturing of human renal progenitors in the VRADD medium resulted in their differentiation into podocytes, as demonstrated by novel expression of nephrin, which is the most specific marker of podocytes (Fig. 2A). Interestingly, differentiation of human renal progenitors into podocytes was associated with a reduction of Notch1-3 protein expression (Fig. 2B) and with a downregulation of the Notch-target genes Hes1 and Hes5 (Fig. 2C), and of the RBP-J-dependent promoter reporter activation (Fig. 2D). The fact that only Hes1 and Hes5 and not other target genes were downregulated is in agreement with observations in other stem/progenitor cell types, which also reported that Notch-related effects on proliferation and/or differentiation can be specifically mediated by Hes1 and/or Hes5 [25–27]. To evaluate the possible role of Notch signaling shutdown in the differentiation of human renal progenitors, cells were exposed to DAPT for 48 hours and expression of podocyte markers was assessed. DAPT treatment induced Notch signaling inhibition, as demonstrated by the decrease in Hes1 mRNA levels (data not shown), but the expression of the podocyte marker nephrin was unaffected (Fig. 2E), thus demonstrating that downregulation of the Notch pathway was not sufficient to induce the differentiation of renal progenitors.

**Figure 3:**
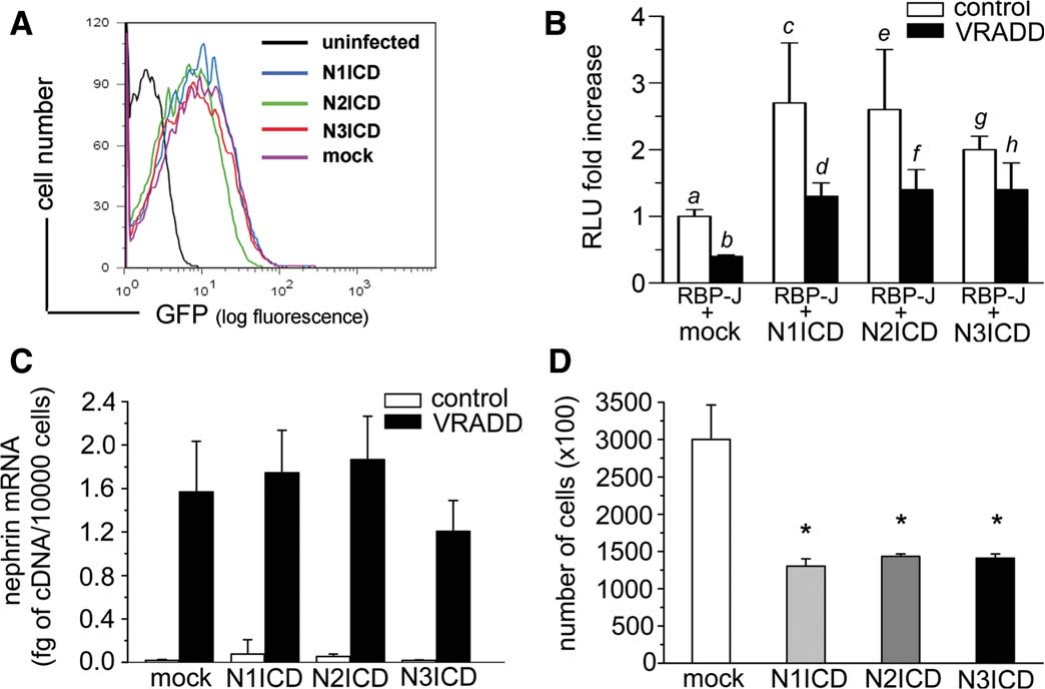
Notch downregulation is necessary to achieve differentiation of human renal progenitors into the podocyte lineage. (**A**): Sustained Notch protein expression in renal progenitors as obtained following infection with a vector leading to the N1ICD, N2ICD, or N3ICD and the GFP to be simultaneously coexpressed. Cells infected with the empty vector (mock) express GFP but do not express the NICDs. Uninfected cells are shown for comparison. One representative of 10 independent experiments is shown. (**B**): Downregulation of the Notch pathway activity following differentiation of renal progenitors toward the podocyte lineage is rescued by infection with vectors expressing N1ICD, N2ICD, or N3ICD as demonstrated by coinfection with a reporter vector for the RBP-J transcriptional response element. Results are expressed as mean ± SEM fold increase of luciferase activity in renal progenitors coinfected with a reporter vector for the RBP-J and the vectors expressing N1ICD, N2ICD, or N3ICD in comparison with the empty vector before (control) or after their differentiation toward the podocyte lineage (VRADD) as obtained in at least four independent experiments. *b* versus *a*, *c*, *d*, *e*, *f*, *g*, *h*: *p* < .05; a versus c, e, g: *p* < .05; *a* versus *d*, *e* versus *f*, and *g* versus *h*: *NS*. (**C**): Analysis of nephrin mRNA fold increase after VRADD culture in renal progenitors infected with an empty vector (mock) and with vectors expressing N1ICD, N2ICD, or N3ICD. Results are expressed as mean ± SEM obtained in four separate experiments. (**D**): Effect of infection with mock vector and vector expressing N1ICD, N2ICD, or N3ICD on cell number after 48 hours of culture in VRADD medium. Results are expressed as mean ± SEM of triplicate assessment as obtained in four separate experiments (*, *p* < .05). Abbreviations: GFP, green fluorescent protein; RBP-J, recombination signal-binding protein-J; RLU, relative luminescence unit; NICD, Notch intracellular domain; NS, not significant; VRADD, vitamin D_3_, retinoic acid and dexamethasone-supplemented DMEM/F12.

To investigate whether the downregulation of Notch signaling was however necessary for the differentiation into the podocyte lineage, we induced a sustained Notch signaling via the infection of renal progenitors with a recombinant lentivirus, which encoded the intracellular domain of the three Notch receptors: N1ICD, N2ICD, or N3ICD (Fig. 3A). Infection of NICDs resulted in an upregulation of RBP-J transactivation (Fig. 3B). Empty vector- (mock) and NICD-infected renal progenitors were then induced to differentiate into podocytes (Fig. 3B). Treatment with VRADD turned off almost completely the Notch pathway in mock-infected progenitors, while it exerted only a minor effect on NICD-infected renal progenitors (Fig. 3B). The differentiation process occurred normally in mock-infected renal progenitors, but it was perturbed in NICD-infected cells (Fig. 3C, 3D). Indeed, most of the NICD-infected renal progenitors detached from the plate and died following differentiation toward the podocyte lineage (Fig. 3D). However, the few surviving cells expressed levels of nephrin as high as those expressed by mock-infected renal progenitors after their differentiation (Fig. 3C). Similar effects were observed independently of the type of NICD infected (Fig. 3C, 3D) and of the level of NICD expression of RBP-J transactivation achieved. Interestingly, an almost complete exhaustion of the Notch activity was required to avoid death of renal progenitors during their differentiation toward the podocyte lineage. Indeed, similar levels of Notch activity had no effect on survival and promoted growth of undifferentiated renal progenitors, but they were incompatible with differentiation. These results suggest that the death-promoting effect of Notch activation in podocytes was cell type-specific and was not related to Notch overexpression.

### Notch Activation Differentially Regulates Cell Cycle Progression and Checkpoint Arrest in Renal Progenitors and Podocytes

**Figure 4:**
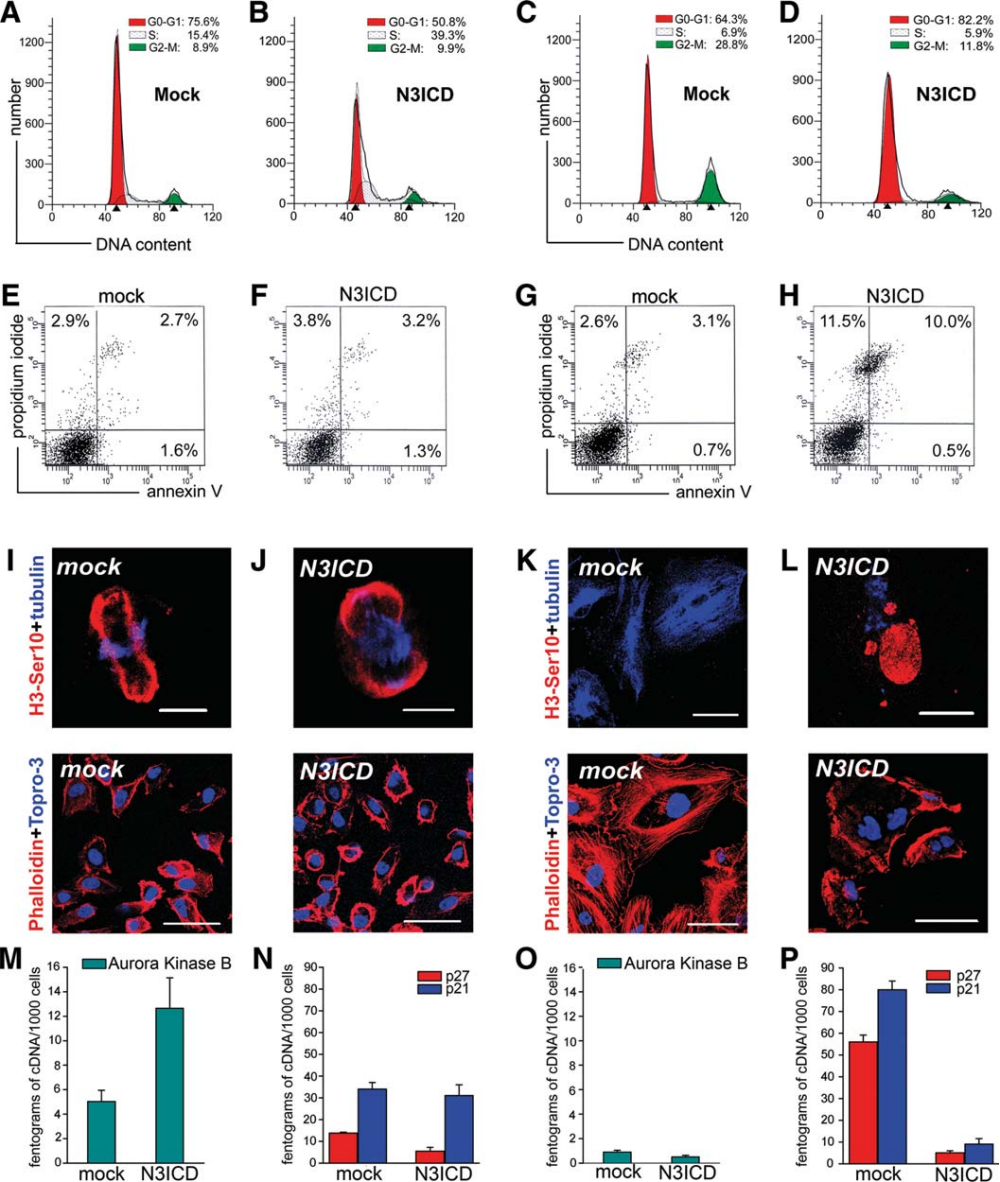
Regulation of cell cycle progression, mitosis, cell death, and cytoskeleton organization in N3ICD-infected human renal progenitors before and after differentiation toward the podocyte lineage. (**A, B**): Cell cycle analysis performed on (**A**) mock- and (**B**) N3ICD-infected renal progenitors. One representative of four independent experiments is shown. (**C, D**): Cell cycle analysis performed on (**C**) mock- and (**D**) N3ICD-infected renal progenitors after their differentiation toward the podocyte lineage. One representative of four independent experiments is shown. (**E, F**): FACS analysis of apoptosis/necrosis in mock- and N3ICD-infected renal progenitors as assessed by annexin-V and propidium iodide (PI) staining. One representative of four independent experiments is shown. (**G, H**): FACS analysis of apoptosis/necrosis in mock- and N3ICD-infected renal progenitors after their differentiation toward the podocyte lineage as assessed by annexin-V and PI staining reveals an increase in the percentage of PI/annexin V positive cells in N3ICD-infected cells. One representative of four independent experiments is shown. (**I, J**): Above: H3-Ser10 (red) and tubulin (blue) staining of mock- and N3ICD-infected undifferentiated renal progenitors reveals normal mitoses. For mock-infected cells, a representative metaphase is shown, while for N3ICD-infected cells, a representative anaphase is shown. One representative of six independent experiments is shown. Scale bar = 10 μm. Below: Phalloidin staining (red) of mock- and N3ICD-infected undifferentiated renal progenitors. Topro-3 (blue) counterstains nuclei. One representative of six independent experiments is shown. Scale bar = 50 μm. (**K, L**): Above: H3-Ser10 (red) and tubulin (blue) staining of renal progenitors infected with vector expressing N3ICD (**L**) after their differentiation toward the podocyte lineage reveals aberrant mitoses characterized by micro/multinucleation (red) and abnormal spindle distribution (blue) in comparison with those infected with an empty vector (mock, [**K**]). One representative of six independent experiments is shown. Scale bar = 10 μm. Below: Phalloidin staining (red) of mock- (**K**) and N3ICD-infected (**L**) renal progenitors after their differentiation toward the podocyte lineage reveals F-actin filaments distributed as stress-like bundles along the axis of the cells in mock-infected podocytes and redistribution of F-actin fibers to the periphery of the cells in podocytes infected with N3ICD. Topro-3 (blue) counterstains nuclei. One representative of six independent experiments is shown. Scale bar = 50 μm. (**M, N**): Assessment by real-time quantitative reverse transcription polymerase chain reaction (RT-PCR) of Aurora kinase B (**M**), p21^Cip1/WAF-1^ and p27^Kip1^ (**N**) mRNA expression in mock- and N3ICD-infected undifferentiated renal progenitors. Results are expressed as mean ± SEM of triplicate assessments in seven separate experiments. (**O, P**): Assessment by real-time quantitative RT-PCR of Aurora kinase B (**O**) and p27^Kip1^, p21^Cip1/WAF-1^ (**P**) mRNA expression in mock- and N3ICD-infected renal progenitors after their differentiation toward the podocyte lineage. Results are expressed as mean ± SEM of triplicate assessments in seven separate experiments. Abbreviation: FACS, fluorescence activated cell sorting.

We then evaluated the cell cycle distribution of mock- and NICD-infected human renal progenitors maintained in undifferentiated conditions or induced to differentiate toward the podocyte lineage. Cells were synchronized by detachment and plating, and immediately after adhesion, medium was changed and renal progenitors were either maintained in basal medium or treated with podocyte differentiating medium. Analysis of the cell cycle distribution after 48 hours demonstrated that in NICD-infected undifferentiated renal progenitors the percentage of cells in the S-phase was significantly increased in comparison with mock-infected cells (36.5 ± 2.7 vs. 19.8 ± 2.5, *p* < .05; Fig. 4A vs. 4B). When mock-infected renal progenitors were induced to differentiate toward the podocyte lineage, they synchronized into the G2/M phase of the cell cycle (30.8 ± 6.5 vs. 8.7 ± 2.6, *p* < .05; Fig. 4C vs. 4A), in agreement with previous observations [28]. However, if NICD-infected renal progenitors were induced to differentiate toward the podocyte lineage, there was a substantial reduction of the number of cells in the G2/M phase (11.7 ± 1.9 vs. 30.8 ± 6.5, *p* < .05; Fig. 4C vs. Fig. 4D) and a concurrent impressive podocyte detachment. Similar effects were observed independently of the type of NICD infected. These results suggest that sustained Notch activation during differentiation toward the podocyte lineage causes an override of the G2/M checkpoint and promotes cell death. To support this possibility, the percentage of apoptotic and/or necrotic progenitors and podocytes in mock- or NICD-infected cells was evaluated by using a combined FACS analysis for PI and annexin V (Fig. 4E–4H). The percentage of dead cells was analyzed including adherent and detached cells floating in the supernatant. Renal progenitors showed a negligible percentage of dead cells, independently of the level of Notch expression (Fig. 4E, 4F). By contrast, following their differentiation toward the podocyte lineage, NICD expression increased the percentage of dead cells among NICD-infected progenitors in comparison with mock-infected progenitors (5.1 ± 0.9 vs. 21.1 ± 2.1, *p* < .05; Fig. 4G vs. 4H). Taken together, these results suggest that Notch activation impairs the progression of podocytes through mitosis. We thus evaluated the appearance of mitotic divisions in renal progenitors before and after their differentiation toward podocytes using double immunofluorescence analysis for the phosphorylated Histone H3-serine 10 (H3-Ser10), a mitosis marker, and the mitotic spindle component tubulin. Normal mitoses were observed in undifferentiated mock-infected renal progenitors, which displayed a conserved cytoskeleton structure and normal nuclear ploidy (Fig. 4I), that were not influenced by infection with NICDs (Fig. 4J). By contrast, following their differentiation toward podocytes, NICD-infected progenitors revealed the presence of aberrant mitoses, binucleated or micronucleated cells and a disrupted cytoskeleton (Fig. 4L) in comparison with their respective mock-infected cells (Fig. 4K), thus suggesting death by mitotic catastrophe.

To establish the reason for the different effect of Notch activation on mitotic division in undifferentiated versus differentiated renal progenitors, we evaluated the mRNA levels of the cell cycle regulators, p21^Cip1/WAF-1^, p27^Kip1^. Aurora kinase B, which plays a critical role in mitotic entry and chromosome segregation, was also evaluated. Infection of renal progenitors with NICDs consistently resulted in the upregulation of transcription of Aurora kinase B (*p* < .05 vs. mock; Fig. 4M), as well as in a downregulation of p27^Kip1^ (*p* < .05 vs. mock; Fig. 4N). By contrast, differentiation of renal progenitors toward the podocyte lineage resulted in a strong downregulation of Aurora kinase B transcription (*p* < .05 Fig. 4O vs. mock in Fig. 4M) and upregulation of p21^Cip1/WAF-1^ and p27^Kip1^ (*p* < .05 Fig. 4P vs. mock in Fig. 4N), which is consistent with the inability of the podocyte to divide. However, in NICD-infected podocytes, a downregulation in the transcription of p21^Cip1/WAF-1^ and p27^Kip1^ in comparison with mock podocytes (*p* < .05 vs. mock; Fig. 4P) was observed, suggesting that progression through the cell cycle could not be arrested. Furthermore, in presence of low levels of Aurora kinase B (*p* < .05 vs. mock; Fig. 4O), cells could not assemble a functional mitotic spindle, suggesting that they overrode the G2/M checkpoint and progressed through an aberrant mitosis. Similar effects were observed independently of the type of NICD infected (Supporting Information Fig. 1 and 2) and of the level of Notch activation achieved. Taken together, these results demonstrate that Notch activation upregulates Aurora Kinase B and induces progression through the cell cycle and cell division in undifferentiated human renal progenitors. By contrast, Notch activation in podocytes, which express irrelevant levels of Aurora kinase B, induces cell cycle progression in cells that cannot assemble a functional mitotic spindle thus causing death by mitotic catastrophe.

### Activation of the Notch Pathway in Renal Progenitors and Podocytes in Patients Affected by Glomerular Disorders Related to Podocyte Injury

**Figure 5:**
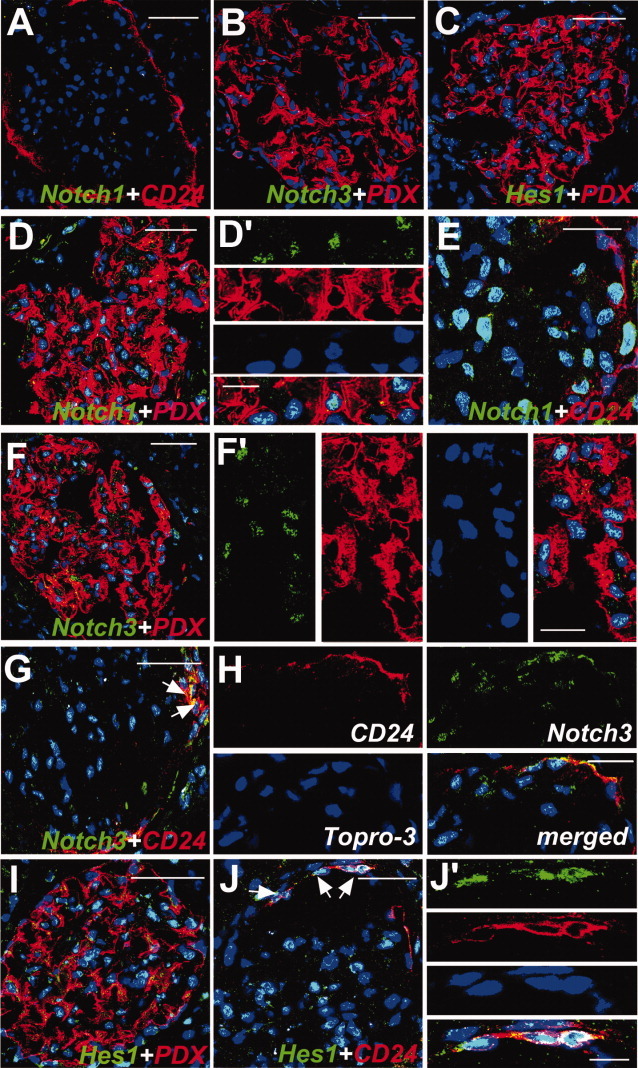
Upregulation of the Notch signaling components in glomeruli of patients affected by LES nephritis or focal segmental glomerulosclerosis (FSGS). (**A**): Double label immunofluorescence for Notch1 (green) and CD24 (red) in healthy adult human kidneys. Scale bar = 50 μm. (**B**): Double label immunofluorescence for Notch3 (green) and PDX (red) in healthy adult human kidneys. Scale bar = 50 μm. (**C**): Double label immunofluorescence for Hes1 (green) and PDX (red) in healthy adult human kidneys. Scale bar = 50 μm. (**D**): Double label immunofluorescence for Notch1 (green) and PDX (red) in a patient affected by LES nephritis. Scale bar = 50 μm. (**D**′): High-power magnification of Notch1+PDX+ cells in the glomerular tuft. Scale bar = 10 μm. (**E**): Double label immunofluorescence for Notch1 (green) and CD24 (red) in a patient affected by FSGS. Scale bar = 20 μm. (**F**): Double label immunofluorescence for Notch3 (green) and PDX (red) in a patient affected by LES nephritis. Scale bar = 50 μm. (**F**′): High-power magnification of Notch3+PDX+ cells in the glomerular tuft. Scale bar = 20 μm. (**G**): Double label immunofluorescence for Notch3 (green) and CD24 (red) in a patient affected by LES nephritis. Scale bar = 50 μm. (**H**): Double label immunofluorescence for Notch3 (green) and CD24 (red) in a patient affected by FSGS. Scale bar = 20 μm. (**I**): Double label immunofluorescence for Hes1 (green) and PDX (red) in a patient affected by LES nephritis. Scale bar = 20 μm. (**J**): Double label immunofluorescence for Hes1 (green) and CD24 (red) in a patient affected by LES nephritis. Scale bar = 50 μm. (**J**′): High-power magnification of Hes1+CD24+ renal progenitors. Scale bar = 10 μm. Confocal microscopy was performed on eight healthy human kidneys, bioptic specimens obtained from 15 patients affected by LES nephritis and 10 patients affected by FSGS. Representative images are shown. Topro-3 counterstains nuclei (blue). Abbreviations: LES, systemic lupus erythematosus; PDX, podocalyxin.

To assess the expression of components of the Notch signaling pathway in healthy and pathologic kidneys, we performed immunofluorescence for Notch1, Notch2, and Notch3 in normal human kidneys or in kidney biopsies from patients affected by different types of glomerular disorders characterized by severe podocyte injury, such as LES nephritis and FSGS (Fig. 5). To evaluate the expression of Notch1, Notch2, and Notch3 in podocytes or renal progenitors, we utilized antibodies that selectively recognized the intracellular domain, thus allowing identification of the cells in which activation of the Notch pathway had occurred. The expression of Notch1 and Notch3 was virtually undetectable in normal kidneys (Fig. 5A, 5B), but clearly evident in nuclei of podocytes within the glomeruli of patients with LES nephritis or FSGS, as demonstrated by double immunolabeling with the podocyte marker PDX (Fig. 5D, 5F). Similar results were observed also for Notch2, although this receptor was expressed at lower level (data not shown). In addition, Notch3 expression was observed in nuclei of renal progenitors, as demonstrated by their costaining with the renal progenitor marker CD24 (Fig. 5G–5H), whereas Notch1 expression was more rarely detected (Fig. 5E). Likewise, the expression of Hes1 was scarce and limited to a few cells within the glomeruli in healthy kidneys (Fig. 5C), but it was strongly expressed in podocytes as well as in renal progenitors of patients with LES nephritis or FSGS (Fig. 5I, 5J).

### Notch Inhibition Differentially Regulates Podocyte Injury and Regeneration in Mice with FSGS

**Figure 6:**
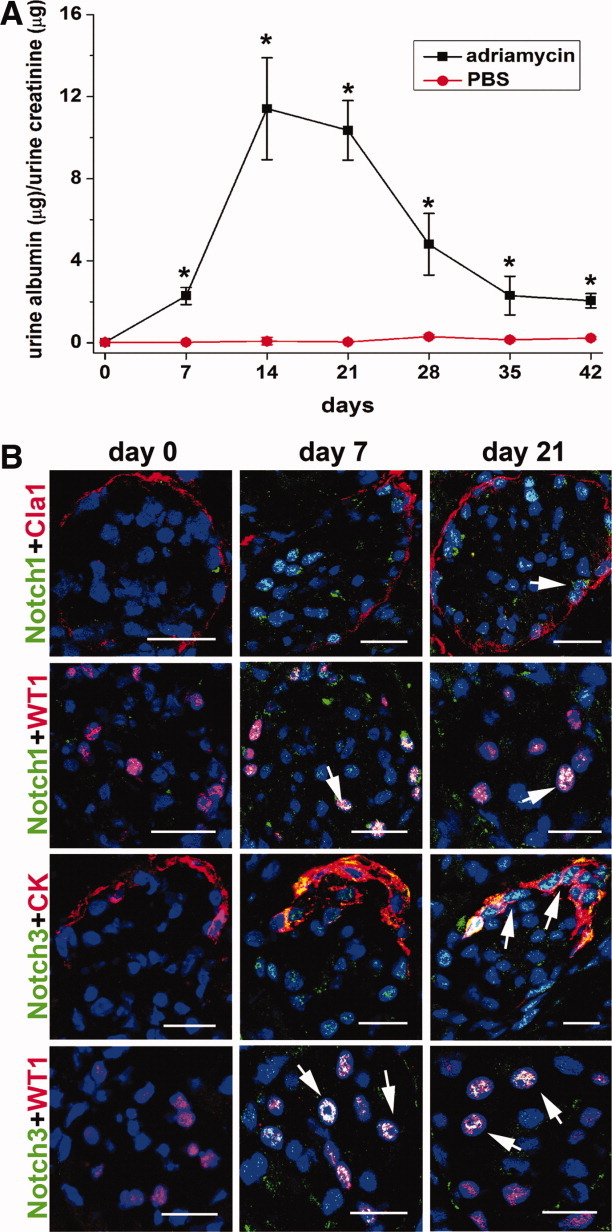
Upregulation of the Notch signaling components in podocytes and/or renal progenitors of SCID mice with adriamycin nephropathy. (**A**): Time course assessment of albumin/creatinine ratio as measured in mice with adriamycin nephropathy (black square) in comparison with PBS-treated mice (red circle). Data are expressed as mean ± SEM as obtained in three independent experiments (*n* = 12 mice at each time point for each group of treatment); *, *p* < .05. (**B**): Double label immunofluorescence for Notch1 (green) and Claudin-1 (Cla1, red); Notch1 (green) and WT1 (red); Notch3 (green) and cytokeratin (CK, red); Notch3 (green) and WT1 (red) in SCID mice affected by adriamycin nephropathy at different time points. Topro-3 counterstains nuclei (blue). One representative of three independent experiments performed on a total of 12 mice is shown. Scale bar = 20 μm. Abbreviations: PBS, phosphate buffered saline; SCID, severe combined immunodeficient.

To evaluate the role of the Notch pathway in the regenerative properties of renal progenitors following podocyte injury in vivo, we used adriamycin-induced nephropathy, which is an experimental analogue of human FSGS [29]. Lymphocytes contribute to renal injury in adriamycin nephropathy [29,30], and the Notch pathway also plays a crucial role in regulating the function of T lymphocytes. Thus, to evaluate the role of the Notch pathway in renal injury and regeneration irrespectively of the interference of T lymphocytes, we established models of adriamycin-induced nephropathy in SCID mice. SCID mice treated with adriamycin developed functional and histological changes similar to those of immunocompetent mice, displaying a significant proteinuria after 7 days, that peaked between 14 and 21 days and then decreased, stabilizing at pathological values after 28 days, when glomerulosclerosis had already developed in many glomeruli (Fig. 6A). Consistently with results obtained in human tissues, Notch activation was not observed in healthy SCID mice (Fig. 6B). Seven days after adriamycin injection, an activation of the Notch1 and Notch3 pathways was observed in podocytes, as demonstrated by double immunofluorescence for Notch1 or Notch3 and the podocyte marker WT1 (Fig. 6B). In contrast, renal progenitors were rarely labeled, as demonstrated by double immunofluorescence for Notch1 or Notch3 and the mouse renal progenitor markers [11,14], Claudin-1 or cytokeratin (Fig. 6B). After 21 days, Notch1 and Notch3 activation in podocytes persisted (Fig. 6B). More importantly, a strong Notch3 activation was observed in renal progenitors (Fig. 6B), while Notch1 activation was more rarely detected (Fig. 6B).

**Figure 7:**
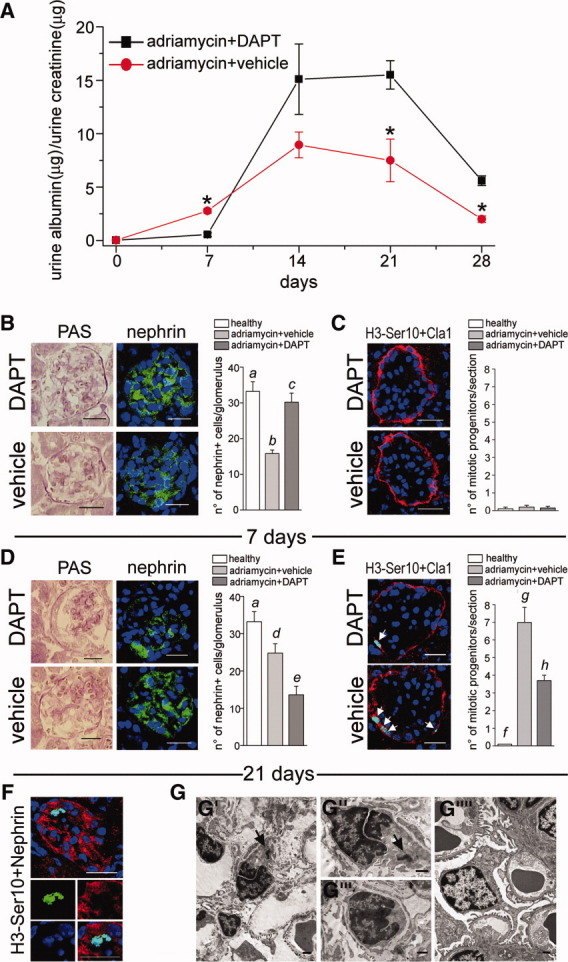
Notch inhibition differentially regulates the balance between podocyte injury and regeneration in mice with focal segmental glomerulosclerosis. (**A**): Time course assessment of albumin/creatinine ratio as measured in mice with adriamycin nephropathy undergoing daily treatment with vehicle (red circle) or with DAPT (black square). Data are mean ± SEM as obtained in three independent experiments (*n* = 15 mice at each time point for each group of treatment); *, *p* < .05. (**B**): Left: PAS staining of renal sections of mice with adriamycin-induced nephropathy treated with vehicle or with DAPT. Scale bar = 20 μm. Middle: Nephrin staining (green) shows significant podocyte depletion in vehicle treated in comparison with DAPT-treated mice. Topro-3 (blue) counterstains nuclei. Scale bar = 20 μm. Right: Quantitation of number of podocytes (nephrin+ cells)/glomerulus is shown. Data are mean ± SEM as obtained in three independent experiments. *a* versus *b*, *a* versus *c*, and *b* versus *c*; *p* < .001. (**C**): Left: Double label immunofluorescence for the mitosis marker H3-Ser10 (green) and the mouse renal progenitor marker Cla1 (red) shows the absence of mitotic renal progenitors after 7 days of injury. Topro-3 (blue) counterstains nuclei. Scale bar = 20 μm. Right: Quantitation of number of mitotic progenitors (H3-Ser10+Cla1+ cells)/section is shown. Data are mean ± SEM as obtained in three independent experiments. (**D**): Left: PAS staining of renal sections of mice with adriamycin-induced nephropathy treated with vehicle or with DAPT. Scale bar = 20 μm. Middle: Nephrin staining (green) shows podocyte depletion in DAPT-treated in comparison with vehicle-treated mice. Topro-3 (blue) counterstains nuclei. Scale bar = 20 μm. Right: Quantitation of number of podocytes (nephrin+ cells)/glomerulus is shown. Data are mean ± SEM as obtained in three independent experiments. *a* versus *d*, *a* versus *e*, *b* versus *d*, and *c* versus *e*; *p* < .001. (**E**): Left: Double label immunofluorescence for H3-Ser10 (green) and Cla1 (red) shows significantly higher numbers of mitotic renal progenitors after 21 days of injury in vehicle treated in comparison with DAPT-treated mice. Topro-3 (blue) counterstains nuclei. Scale bar = 20 μm. Right: Quantitation of number of mitotic progenitors (H3-Ser10+Cla1+ cells)/section is shown. Data are mean ± SEM as obtained in three independent experiments. *f* versus *g*, *f* versus *h*, and *g* versus *h*; *p* < .001. (**F**): H3-Ser10 (green) and nephrin (red) shows the occurrence of mitotic podocytes with abnormal nuclear morphology. Topro-3 (blue) counterstains nuclei. Scale bar = 20 μm. A representative image is shown. (**G**): Electron microscopy reveals aberrant mitoses. (G′): Electron micrographs of glomerular podocytes from a mouse with adriamycin nephropathy showing abnormal nuclei and a micronucleus (arrow) consistent with mitotic catastrophe. (G″): High-power magnification of (G′) better depicting the micronucleus (arrow). (G″′): High-power magnification of a heterochromatic nucleus with peculiar symmetrical chromatin pattern, reminiscent of aberrant mitosis. (G″″): A normal podocyte is shown for comparison. Scale bar = 1 μm. Representative images are shown. Abbreviations: DAPT, N-[N-(3,5-difluorophenacetyl)-L-alanyl]-S-phenylglycine t-butyl ester; PAS, periodic acid schiff.

To evaluate the role of the Notch pathway in renal injury and regeneration, SCID mice affected by adriamycin-induced nephropathy were treated with DAPT or its vehicle. Mice with adriamycin-induced nephropathy showed high urinary albumin/creatinine ratio levels that were unaffected by injection of vehicle (Fig. 7A). By contrast, injection of DAPT induced a reduction of proteinuria after 7 days of treatment, while at later time points mice treated with DAPT showed a worsening of renal proteinuria, as reflected by significantly higher urinary albumin/creatinine ratios (Fig. 7A). Interestingly, the effects of DAPT were enhanced when severe proteinuria occurred. In acute kidney injury (day 7), the improvement of proteinuria induced by injection of DAPT was associated with reduced glomerular injury, as demonstrated in periodic acid schiff (PAS)-stained sections (Fig. 7B). Accordingly, in vehicle-treated mice there was a decrease in the number of podocytes, identified as nephrin-expressing cells, whereas in DAPT-treated mice podocytes were preserved (Fig. 7B). However, double label immunofluorescence for H3-Ser10 and Claudin-1 proliferating progenitors were very rare, suggesting that the regenerative response had not started yet (Fig. 7C). By contrast, in chronically injured kidneys (day 21), the worsening of proteinuria induced by injection of DAPT was associated with increased glomerular injury, as demonstrated in PAS-stained sections in comparison with vehicle-treated mice (Fig. 7D). Consistently, in DAPT-treated mice the number of podocytes was decreased (Fig. 7D), whereas in vehicle-treated mice the number of podocytes was significantly increased and consistently higher than the number of podocytes observed in vehicle-treated mice at day 7. These findings suggest that on day 21, in vehicle-treated mice new podocytes had been regenerated and podocyte injury was partially repaired (Fig. 7D). Accordingly, on day 21, double label immunofluorescence for H3-Ser10 and Claudin-1 demonstrated the presence of numerous proliferating renal progenitors within the Bowman's capsule in vehicle-treated mice (Fig. 7E), suggesting an ongoing regenerative response of renal progenitors. Strikingly, in DAPT-treated mice, there was a reduction in the number of proliferating renal progenitors in comparison with vehicle-treated mice (Fig. 7E). To further characterize the response to podocyte injury, double label immunofluorescence for H3-Ser10 and nephrin (Fig. 7F) was performed. In mice affected by adriamycin-induced nephropathy, some podocytes in mitosis were observed at all the time points analyzed (Fig. 7F). However, mitotic podocytes were often characterized by abnormal H3-Ser10 distribution and sometimes by micro/multinucleation, suggesting that they were undergoing abnormal mitosis. To support this hypothesis, electron microscopy was performed, demonstrating that proliferating podocytes were undergoing aberrant mitosis (Fig. 7G). To address the frequency of this event, the number of mitoses observed in podocytes was quantified at different time points. In vehicle-treated mice with adriamycin nephropathy, mitotic podocytes were frequently observed, their number being higher at earlier time points (3.1 ± 0.3/glomerulus at day 7 vs. 2.3 ± 0.2/glomerulus at day 21: *p* < .05). More than 70% of the mitotic figures showed major aberrations at all time points analyzed (73.8% ± 18.3% at day 7 vs. 71.5% ± 14.1% at day 21: *NS*), thus confirming that mitosis in podocytes represented a death event. Consistently, DAPT treatment reduced the number of mitoses in podocytes (3.1 ± 0.3/glomerulus vs. 1.4 ± 0.3/glomerulus at day 7: *p* < .05; 2.3 ± 0.2/glomerulus vs. 1.1 ± 0.3/glomerulus at day 21: *p* < .05).

## DISCUSSION

The molecular mechanisms regulating the proliferation of renal progenitors, as well as the cell fate determination in the podocyte lineage are unknown. Here, we demonstrate the role of the Notch signaling pathway in both these processes.

The first important finding emerging from this study was that Notch activation triggers the expansion of renal progenitors by promoting their entry into the S-phase of the cell cycle and mitotic division. This represents the first demonstration of a signaling pathway that can promote the regenerative capacity of renal progenitor cells. Interestingly, an attenuation of Notch activity was observed during the commitment of renal progenitors to the podocyte cell fate. However, Notch downregulation was neither sufficient nor necessary for the acquisition of a podocyte phenotype, but an impaired downregulation of the Notch pathway led to podocyte death. Indeed, renal progenitor differentiation into podocytes was associated with cell cycle checkpoint activation and G_2_/M arrest, reflecting an intrinsic barrier to replication of mature podocytes. Persistent activation of the Notch pathway induced podocytes to cross the G_2_/M checkpoint, resulting in cytoskeleton disruption and cell death.

The pivotal role of podocyte depletion in glomerulosclerosis has been established [1–5]. However, the mechanisms responsible for podocyte loss in response to injury are still mostly unknown. Some authors have suggested that podocytes might be lost because of apoptosis and/or cell detachment, but in several glomerular disorders apoptotic podocytes were not observed, suggesting that different mechanisms of cell death may be involved [5,31–33]. In this study, we provide the first evidence that podocytes can die by mitotic catastrophe induced by Notch activation. Mitotic catastrophe is a cell death mode occurring either during or shortly after a dysregulated/failed mitosis and can be accompanied by morphological alterations including micro/multinucleation, which is the only sign that distinguishes mitotic catastrophe from either apoptosis or necrosis [34,35]. Although mitotic catastrophe was not previously described as a mechanism of podocyte death, there is evidence to suggest that podocytes undergo catastrophic mitosis in glomerular disorders. Indeed, the presence of multinucleated podocytes has been nonspecifically reported in a variety of experimental [36–39] as well as human [40–43] glomerulopathies.

The results of the present study also suggest that podocytes can initiate DNA synthesis, but cannot undergo cytokinesis. Indeed, cell division appears to be possible only for renal progenitors, which display a cuboidal/flat cell shape with simple cytoskeleton architecture that allows progression through mitosis and proliferation. As soon as renal progenitors differentiate into the octopus-like phenotype of the mature podocyte, they lose their ability for cell multiplication. Indeed, on re-entering the cell cycle podocytes would destroy the specifically arranged cytoskeletal organization [44–46], including actin systems, which is essential to maintain their integrity and permselectivity. Consistently, renal progenitor differentiation toward the podocyte lineage is characterized by upregulation of the p21^Cip1/WAF-1^ and p27^Kip1^ inhibitors, which impair the capacity of these cells to progress through the cell cycle. Downregulation of p21^Cip1/WAF-1^ and p27^Kip1^ induced by Notch activation forces progression toward mitosis of a cell that cannot assemble an efficient mitotic spindle, because it poorly expresses Aurora kinase B, which is essential for cytokinesis. Taken altogether, these results suggest that entry into mitosis for podocytes is a trigger for death, thus explaining why podocytes cannot be replaced through division of adjacent podocytes. However, even glomerular disorders with severe injury can undergo regression and remission, suggesting that podocyte loss can be rescued [47,48].

The discovery of renal progenitors [6] as well as of their ability to generate novel podocytes [10,11] are consistent with the observation provided in this study that the outcome of glomerular disorders depends on a balance between podocyte injury and regeneration provided by renal progenitors. These results raise some additive questions. For example, which may be the stimulus(i) that induce Notch upregulation in podocytes and/or progenitors. Previous studies demonstrated that transforming growth factor-beta (TGF-β) [49,50], which is released following podocyte injury or proteinuria [51], is a powerful Notch activator, suggesting that Notch activation may represent an adaptive response to podocyte injury. Interestingly, although the biological effects of distinct Notch receptors appeared similar, a differential expression of Notch receptor subtypes was observed in renal progenitors or podocytes in FSGS and in LES nephritis, Notch3 being the prevalent Notch receptor subtype upregulated in renal progenitors. These results suggest that a differential modulation of injury and regeneration through distinct Notch receptors may occur. Further studies are required to address this point.

Finally, our results provide an explanation for the observations of two previous studies, showing that transgenic mice carrying a specific activation of the N1ICD in podocytes displayed chronic glomerular injury with albuminuria [50,52]. However, while active Notch1 expression induced podocyte death in one study [50], it led to proliferation of cells with an immature podocyte phenotype within glomeruli in the other [52]. The results of our study allow to reconcile these apparently contradictory observations. Indeed, these two studies differed for the timing of Notch1 expression, suggesting that while active Notch1 expression induced death in mature podocytes, it led to their proliferation if expressed before their full differentiation [53]. According to our results, Notch activation in podocyte progenitors induces proliferation, thus suggesting that in the study by Waters et al. [52], podocyte injury induced by Notch activation in differentiated podocytes may have been partially overcome by Notch activation in podocyte progenitors, explaining the milder proteinuria and less severe renal failure in comparison with Niranjan et al. [50].

## CONCLUSION

Taken altogether, the results of this study provide the first demonstration that the severity of glomerular disorders depends on the Notch pathway-regulated balance between the degree of podocyte injury and the amount of regeneration provided by renal progenitors, thus opening the previously unthinkable possibility that in patients with glomerular disorders this balance might be manipulated to trigger podocyte regeneration.

## Disclosure of Potential Conflicts of Interest

The authors indicate no potential conflicts of interest.
