# Supplementary material for: Notch Activation Differentially Regulates Renal Progenitors Proliferation and Differentiation Toward the Podocyte Lineage in Glomerular Disorders
Source: Stem Cells. 2010 Aug 2;28(9):1674–85. doi: 10.1002/stem.492 (PMC2996085; doi:10.1002/stem.492)
Supplement: Supplementary file 1 [file stem0028-1674-SD1.doc]

**Supplementary Figures**

**
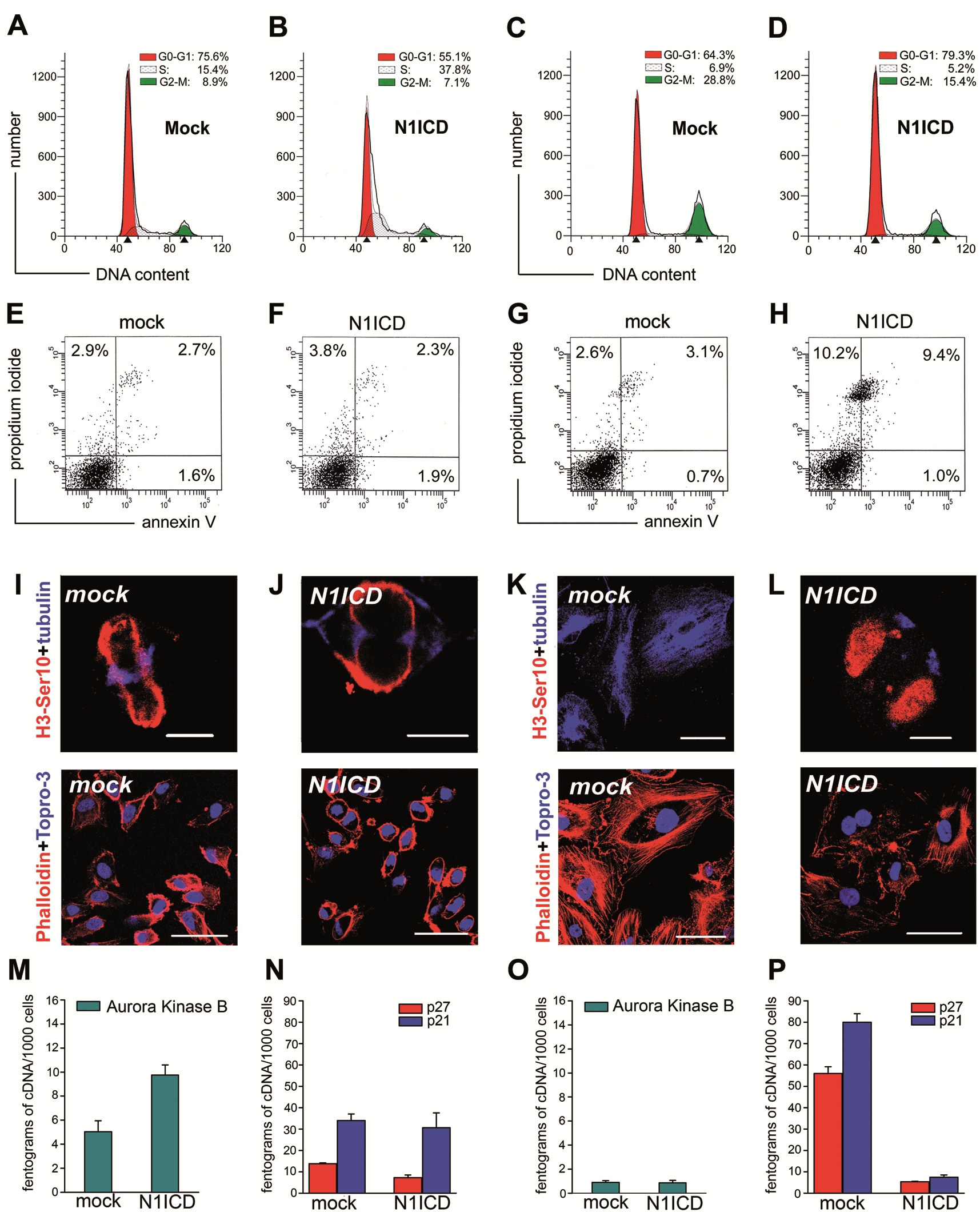
**

**Supplementary Figure 1. Regulation of cell cycle progression, mitosis, cell death and cytoskeleton organization in N1ICD-infected renal progenitors before and after differentiation toward the podocyte lineage**

(A-B) Cell cycle analysis performed on (A) mock- and (B) N1ICD-infected renal progenitor cells. One representative experiment is shown. (C-D) Cell cycle analysis performed on (C) mock-, and (D) N1ICD-infected renal progenitor cells after their differentiation toward the podocyte lineage. One representative of four experiments is shown. (E-F) FACS analysis of apoptosis/necrosis in renal progenitor cells infected with an empty vector (mock) and with vectors expressing N1ICD as assessed by annexin-V and PI staining. One representative of four experiments is shown. (G-H) FACS analysis of apoptosis/necrosis in renal progenitors infected with an empty vector (mock) and with vectors expressing N1ICD after their differentiation toward the podocyte lineage as assessed by annexin-V and PI staining reveals an increase in the percentage of PI/annexin V positive cells in NICD-infected cells. One representative experiment of four independent experiments is shown. (I-J) Above: H3-Ser10 (red) and tubulin (blue) staining of undifferentiated renal progenitor cells infected with an empty vector (mock) and with vectors expressing N1ICD reveals normal mitoses. For mock- as well as for N1ICD-infected cells a representative metaphase is shown. One representative of six experiments is shown. Bar 10 μm. Below: Phalloidin staining (red) of undifferentiated renal progenitor cells infected with an empty vector (mock) and with vectors expressing N1ICD. Topro-3 (blue) counterstains nuclei. One representative of six experiments is shown. Bar 50 μm. (K-L) Above: H3-Ser10 (red) and tubulin (blue) staining of renal progenitor cells infected with vectors expressing N1ICD (L) after their differentiation toward the podocyte lineage reveals aberrant mitoses characterized by micronucleation and abnormal spindle distribution in comparison with those infected with an empty vector (mock, K). One representative of six experiments is shown. Bar 10 μm. Below: Phalloidin staining (red) of renal progenitors cells infected with an empty vector (mock, K) and with vectors expressing N1ICD (L) after their differentiation toward the podocyte lineage reveals F-actin filaments distributed as stress-like bundles along the axis of the cells in mock-infected podocytes and redistribution of F-actin fibers to the periphery of the cells in podocytes infected with N1ICD. Topro-3 (blue) counterstains nuclei. One representative of six experiments is shown. Bar 50 μm. (M-N) Assessment by real-time quantitative RT-PCR of Aurora kinase B (M) and p27, p21 (N) mRNA expression in undifferentiated renal progenitor cells infected with an empty vector (mock) and with vectors expressing N1ICD. Results are expressed as mean ± SEM of triplicate assessments in seven separate experiments. (O-P) Assessment by real-time quantitative RT-PCR of Aurora kinase B (O), and p27, p21 (P) mRNA expression in renal progenitor cells after their differentiation toward the podocyte lineage infected with an empty vector (mock) and with vectors expressing N1ICD. Results are expressed as mean ± SEM of triplicate assessments in seven separate experiments.

**
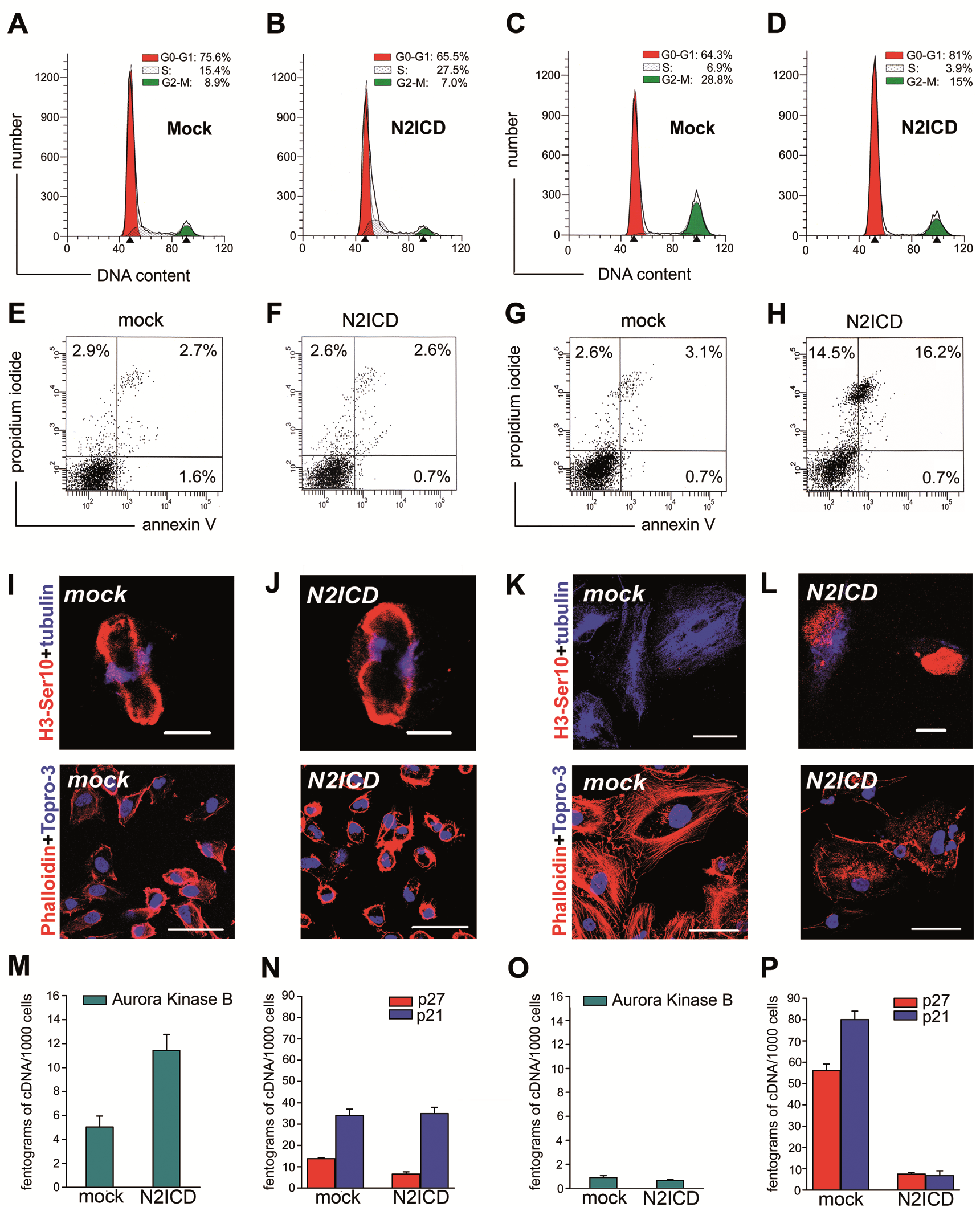
**

**Supplementary Figure 2. Regulation of cell cycle progression, mitosis, cell death and cytoskeleton organization in N2ICD-infected renal progenitors before and after differentiation toward the podocyte lineage**

(A-B) Cell cycle analysis performed on (A) mock- and (B) N2ICD-infected renal progenitor cells. One representative experiment is shown. (C-D) Cell cycle analysis performed on (C) mock-, and (D) N2ICD-infected renal progenitor cells after their differentiation toward the podocyte lineage. One representative of four experiments is shown. (E-F) FACS analysis of apoptosis/necrosis in renal progenitor cells infected with an empty vector (mock) and with vectors expressing N2ICD as assessed by annexin-V and PI staining. One representative of four experiments is shown. (G-H) FACS analysis of apoptosis/necrosis in renal progenitors infected with an empty vector (mock) and with vectors expressing N2ICD after their differentiation toward the podocyte lineage as assessed by annexin-V and PI staining reveals an increase in the percentage of PI/annexin V positive cells in NICD-infected cells. One representative experiment of four independent experiments is shown. (I-J) Above: H3-Ser10 (red) and tubulin (blue) staining of undifferentiated renal progenitor cells infected with an empty vector (mock) and with vectors expressing N2ICD reveals normal mitoses. For mock- as well as for N1ICD-infected cells a representative metaphase is shown. One representative of six experiments is shown. Bar 10 μm. Below: Phalloidin staining (red) of undifferentiated renal progenitor cells infected with an empty vector (mock) and with vectors expressing N2ICD. Topro-3 (blue) counterstains nuclei. One representative of six experiments is shown. Bar 50 μm. (K-L) Above: H3-Ser10 (red) and tubulin (blue) staining of renal progenitor cells infected with vectors expressing N2ICD (L) after their differentiation toward the podocyte lineage reveals aberrant mitoses characterized by micronucleation and abnormal spindle distribution in comparison with those infected with an empty vector (mock, K). One representative of six experiments is shown. Bar 10 μm. Below: Phalloidin staining (red) of renal progenitors cells infected with an empty vector (mock, K) and with vectors expressing N2ICD (L) after their differentiation toward the podocyte lineage reveals F-actin filaments distributed as stress-like bundles along the axis of the cells in mock-infected podocytes and redistribution of F-actin fibers to the periphery of the cells in podocytes infected with N2ICD. Topro-3 (blue) counterstains nuclei. One representative of six experiments is shown. Bar 50 μm. (M-N) Assessment by real-time quantitative RT-PCR of Aurora kinase B (M), p21^Cip1/WAF-1^ and p27^Kip1^ (N) mRNA expression in undifferentiated renal progenitor cells infected with an empty vector (mock) and with vectors expressing N2ICD. Results are expressed as mean ± SEM of triplicate assessments in seven separate experiments. (O-P) Assessment by real-time quantitative RT-PCR of Aurora kinase B (O) and p27^Kip1^, p21^Cip1/WAF-1^ (P) mRNA expression in renal progenitor cells after their differentiation toward the podocyte lineage infected with an empty vector (mock) and with vectors expressing N2ICD. Results are expressed as mean ± SEM of triplicate assessments in seven separate experiments.
